# ALPK1 agonists: a new strategy for cancer immunotherapy

**DOI:** 10.1038/s41392-026-02659-8

**Published:** 2026-04-24

**Authors:** Michelle C. C. Lim, Gunter Maubach, Michael Naumann

**Affiliations:** Otto von Guericke University, Institute of Experimental Internal Medicine, Medical Faculty, Magdeburg, Germany

**Keywords:** Drug development, Infection

**Fig. 1:**
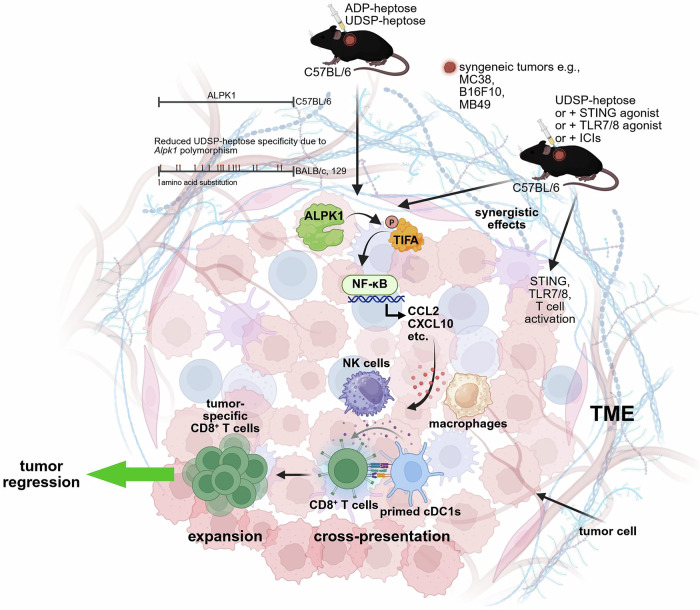
ALPK1-dependent innate immune activation by bacterial metabolites drives antitumor immunity. ALPK1 activation with ADP-heptose, and even more potently with the phosphorothioate analog UDSP-heptose, triggers strong immune responses against tumors. Polymorphisms in *Alpk1* distinguish C57BL/6 mice from BALB/c and 129 mice. The ALPK1 proteins encoded in the BALB/c and 129 strains are identical but differ from that of C57BL/6 by 17 amino acid residues, which reduces specificity for UDSP-heptose. Therefore, it is important to use C57BL/6 mice in developing ALPK1-targeted agonists with therapeutic potential. Intratumoral administration of ADP- or UDSP-heptose in syngeneic mouse models induces ALPK1-dependent phosphorylation of tumor necrosis factor receptor-associated factor-interacting protein with forkhead-associated domain-containing protein A (TIFA) dimers, which in turn triggers the NF-κB signaling cascade within the TME. This induces expression of proinflammatory cytokines and chemokines, including CCL2 and CXCL10, promoting recruitment and activation of innate immune cells such as macrophages and NK cells. Activated conventional type 1 DCs (cDC1s) enhance cross-presentation, resulting in the priming and expansion of tumor-specific CD8⁺ T cells, and subsequent tumor regression. The antitumor mechanism of UDSP-heptose is differentiated from that of TLR7/8 or STING agonists. Nevertheless, synergistic effects were observed upon combination. UDSP-heptose also synergized with ICIs, restoring responsiveness in tumors resistant to single-agent therapy. Created with BioRender.com

A recent study by Tian et al.^1^ in *Nature* shows that alpha-protein kinase 1 (ALPK1) activation with ADP-glycero-β-D-manno-heptose (ADP-heptose), and even more potently with the phosphorothioate analog UDSP-heptose, triggers strong immune responses against tumors, leading to tumor regression through a mechanism distinct from Toll-like receptors 7/8 (TLR7/8) or stimulator of interferon genes (STING) agonists yet capable of synergizing with them when combined (Fig. 1). These findings support a novel therapeutic paradigm that positions ALPK1 agonists as promising targets for next-generation immunotherapy.

Considered a breakthrough in cancer treatment, immunotherapy, including immune checkpoint inhibitors (ICIs) and adoptive T cell therapies, leverages the immune system to recognize and eliminate cancer cells by attenuating immunosuppressive signals of the tumor microenvironment (TME) and amplifying patient T cell-mediated response. However, patient responses are highly heterogeneous, highlighting the need to enhance antitumor immunity by different means.^2^ One approach is targeting the innate immune response, which alters the immune landscape including TME by activating the secretion of interferons, proinflammatory cytokines, and chemokines. Agonists of classical innate immune signaling pathways, such as TLRs and STING, trigger inflammatory cascades that promote immune cell infiltration into tumors and activate adaptive immune responses.^2^ However, their clinical translation has been hampered by dose-limiting toxicity, systemic inflammation, and, particularly in the case of STING agonists, induction of T cell apoptosis and loss of immune durability. Tian et al.^1^ now identify the innate immune cytosolic receptor ALPK1 as a potential strategy to overcome these limitations. Engagement of ALPK1 by ADP-heptose triggers activation of the transcription factor nuclear factor kappa-light-chain-enhancer of activated B cells (NF-κB).^3^ It has also been shown that ALPK1 can be activated allosterically by CDP-heptose and UDP-heptose.^4^ Disease-causing mutations in human ALPK1 show alterations in its specificity for these effectors, leading to aberrant activation by endogenous nucleoside diphosphate (NDP) sugars (GDP-mannose, UDP-mannose, and ADP-ribose), potentially triggering subliminal inflammation.^5^

Tian et al.^1^ showed that administration of ADP-heptose increases proinflammatory chemokines, e.g., CXCL10, CCL2, and CCL4, in mouse and cell culture studies. Additionally, in different mouse tumor models (B16F10-OVA, 4T1-OVA, MC38, and Hepa1-6), intratumoral injections of ADP-heptose inhibited tumor growth significantly. This antitumor effect was absent in *Alpk1*^*−/−*^ mice, indicating that tumor regression is mediated by ALPK1-dependent processes. These data also suggest that tumor cell-intrinsic ALPK1 is not contributing to the observed regression. Building on this therapeutic potential of ALPK1 agonists, different ADP-heptose analogs generated through medicinal chemistry were examined. The phosphorothioate analog of ADP-heptose, UDSP-heptose, proved to be the most potent inducer of NF-κB activation in a reporter assay. Furthermore, UDSP-heptose elicited stronger chemokine induction and tumor regression at lower doses, establishing it as a more potent ALPK1 agonist than ADP-heptose. Notably, the increased potency of UDSP-heptose is attributable to its higher stability, not to enhanced efficiency in ALPK1 activation. Strikingly, mice achieving tumor clearance with UDSP-heptose showed complete resistance to a secondary tumor challenge in the Hepa1-6 model. Thus, ALPK1 activation may promote the formation of immunological memory, suggesting long-term protection against tumor recurrence. Consistent with this notion, ALPK1 activation enhanced the generation of memory CD8⁺ T cells, as evidenced by the marked increase in the proportion of TCF1^+^ progenitor exhausted CD8^+^ T cells among the tumor-infiltrating lymphocytes within the TME of UDSP-heptose-treated tumors. Notably, *Alpk1* is polymorphic, and functional analyses in three mouse strains (C57BL/6, BALB/c, and 129) revealed that UDSP-heptose discriminates among different *Alpk1* allelic variants, underscoring the importance of using C57BL/6 mice in developing ALPK1-targeted agonists with therapeutic potential. Mutations in ALPK1 cause both spiradenoma and retinal dystrophy, optic nerve edema, splenomegaly, anhidrosis, and headache (ROSAH syndrome).^5^ From this perspective, an outstanding question is whether human *ALPK1* polymorphisms affect the efficacy of ALPK1-targeted agonists.

The authors consistently observed induction of the chemokines CXCL10 and CCL2 upon treatment by ALPK1 agonists. Both chemokines are regulated by ALPK1-driven NF-κB activation, although CXCL10 expression typically requires cooperative signaling with interferon-regulated transcription factors. Pharmacological intervention of CXCL10 and CCL2 signaling abolished the antitumor effects of UDSP-heptose, indicating that ALPK1-stimulated antitumor immunity is dependent on these chemokines. CXCL10 and CCL2 are known to recruit lymphocytes and monocytes, respectively, which are required, as bone marrow chimera assays showed that hematopoietic *Alpk1* alone was insufficient to elicit this response. Analyses of TME in UDSP-heptose-treated tumors revealed a significant enrichment of tumor-specific T and natural killer (NK) cell populations. Of particular importance are CD8^+^ T cells, as depletion studies demonstrated that CD8⁺ T cells, but not CD4⁺ T cells or NK cells, mediate the antitumor effects of UDSP-heptose. Mechanistically, ALPK1 agonist-stimulated macrophages and dendritic cells (DCs) promote tumor regression, with DCs performing cross-presentation and driving the expansion of tumor-specific CD8⁺ T cells in tumor-draining lymph nodes. However, the precise ALPK1-expressing cell populations responsible for initiating this response remain unclear. Addressing this question will require cell-specific ALPK1-knockout models, which could reveal the key contributing cell types, how this might vary across tumor types, and guide the development of targeted therapeutic strategies.

Comparison of UDSP-heptose with TLR7/8 and STING agonists revealed mechanistic differences in the immune response. ALPK1 activation induced CXCL10 levels comparable to TLR7/8 or STING signaling but elicited lower IL-6 and TNF production, did not promote T cell apoptosis, and increased the proportion of tumor-specific memory T cells. These features likely underlie the synergistic antitumor effects observed when UDSP-heptose is combined with TLR7/8 or STING agonists. UDSP-heptose also synergized with ICIs, restoring responsiveness in tumors resistant to single-agent therapy. Collectively, these findings highlight the therapeutic relevance of ALPK1 agonists for improving cancer treatment outcomes.

Based on insights from this study, notable questions for future investigation emerge. Do host microbiota and their metabolites modulate ALPK1 signaling, influencing ALPK1 agonist-based therapy efficacy and, more broadly, cancer risk through chronic inflammation? Could engineered oncolytic bacteria provide a platform for the precise delivery of cancer therapeutics such as ALPK1 agonists, restricting ALPK1 activation to the TME? Importantly, the study by Tian et al.^1^ supports the translational relevance of ALPK1 agonists in cancer immunotherapy. This potential is underscored by the recent initiation of a phase 1/2a clinical trial of the ALPK1 activator PTT-936, administered alone or in combination with anti-PD-1/L1 therapy, in patients with solid tumors (NCT06244992).
